# Genetic validation of ABI3 p.Ser209Phe variant and its effects on early brain pathology in asymptomatic elderly individuals

**DOI:** 10.1186/s13195-026-01984-y

**Published:** 2026-02-19

**Authors:** Mikko Koivumäki, Henna Martiskainen, Mari Takalo, Jenni Lehtisalo, Tiia Ngandu, FinnGen, Anniina Snellman, Mikko Hiltunen, Juha O. Rinne

**Affiliations:** 1 Turku PET Centre, University of Turku, Turku, Finland; 2 Turku PET Centre, Turku University Hospital, Turku, Finland; 3 Institute of Biomedicine, University of Eastern Finland, Kuopio, Finland; 4 Department of Public Health, Lifestyles and Living Environments Unit, Finnish Institute for Health and Welfare (THL), Helsinki, Finland; 5 Institute of Clinical Medicine/Neurology, University of Eastern Finland, Kuopio, Finland; 6 Division of Clinical Geriatrics, Center for Alzheimer Research, Care Sciences and Society (NVS), Karolinska Institutet, Stockholm, Sweden; 7 InFLAMES Research Flagship Center, University of Turku, Turku, Finland

**Keywords:** Alzheimer’s disease, ABI3^S209F^, *APOE* ε4, Microglia, β-amyloid-, PET imaging, FinnGen

## Abstract

**Background:**

Alzheimer’s disease (AD) has a strong genetic component, with *APOE* ε4 being the most established risk factor through its effects on beta-amyloid (Aβ) metabolism and microglial function. Recent genetic studies have also implicated microglial genes, such as the ABI3^S209F^ variant, to increased AD risk. As *APOE* ε4 and ABI3^S209F^ influence microglial pathways through distinct mechanisms, their combined analysis may provide novel insights into AD pathophysiology. Therefore, we investigated ABI3^S209F^ in the Finnish FinnGen cohort and in an imaging study of cognitively healthy older adults.

**Methods:**

We used FinnGen R12 data (> 500,000 individuals), including 8,490 ABI3^S209F^ carriers and 511,670 non-carriers, with survival analyses matched by sex and birth year. Disease endpoints (AD, dementia, neurodegenerative disorder) were defined from national health registries using harmonized ICD codes, medication, and reimbursement records. For the imaging study, 58 participants aged ≥ 50 years were recruited into three genotype-based groups (ABI3^S209F^/*APOE* ε4, ABI3^S209F^/*APOE* ε3, non-carriers). All imaging participants underwent structural MRI, [^11^C]PiB PET for amyloid beta, [^11^C]PK11195 PET for microglial activity, and a comprehensive neuropsychological battery.

**Results:**

ABI3^S209F^ was significantly associated with increased risk of AD (OR = 1.22, *p* = 0.0012) and neurodegenerative disorders (OR = 1.21, *p* = 0.00023), but not with dementia (OR = 1.10, *p* = 0.06). Survival analyses indicated that ABI3^S209F^ carriers developed AD at an earlier age than non-carriers with the same *APOE* genotype. The carriers of ABI3^S209F^ and *APOE* ε4 had higher brain Aβ burden when compared to the ABI3^S209F^ carriers without *APOE* ε4 (SUVR 2.0 (0.7) vs. 1.67 (0.5); mean (sd), *p* = 0.017), but there was no difference in Aβ between the ABI3^S209F^ carriers and controls (1.67 (0.5) vs 1.75 (0.6), *p* = 0.75 (HST)). ABI3^S209F^ was not associated with global neuroinflammation, although subtle regional increases in [^11^C]PK11195 binding were observed in ABI3^S209F^ ε4 carriers. No differences were found in brain volumes or cognition.

**Conclusions:**

ABI3^S209F^ increases AD risk and is associated with earlier disease onset. The variant alone does not significantly influence cortical Aβ deposition, neuroinflammation, or brain structure. Its effect may be pronounced in combination with APOEε4.

**Supplementary Information:**

The online version contains supplementary material available at 10.1186/s13195-026-01984-y.

## Introduction

Alzheimer’s disease (AD) has a strong genetic component, with the Apolipoprotein E ε4 (APOEε4) being the most common and well-established genetic risk factor [9, 18]. APOEε4 is associated with a higher burden of beta-amyloid (Aβ) plaques in the brain in a dose-dependent manner [5, 37], and it is thought to impair Aβ clearance [48].

In recent years, genome-wide association studies (GWAS) have identified several additional risk genes for AD [3, 22, 52], including ones with strong expression in microglia, such as *ABI3*. The ABI3 p.Ser209Phe variant (ABI3^S209F^) was first linked to increased AD risk in 2017 with an odds ratio (OR) of 1.43 [41], and this association has been replicated in multiple cohorts [8, 10, 35, 52].

ABI3 is highly expressed in microglial cells, with limited expression in neurons and other glial cell types [8, 39]. Microglia have dual roles in AD pathology, initially protective through Aβ clearance, but later potentially harmful due to sustained inflammation [14, 30]. ABI3 is upregulated in the cortex of AD patients and in Aβ mouse models [6], and is thought to modulate microglial responses through interferon signalling [41], and actin cytoskeletal reorganization via the WAVE2 complex [11, 40]. However, findings from murine Abi3 knockout models have been inconsistent as some show increased Aβ deposition [24, 25], while others note a transient reduction [20]. Despite the replicated association between ABI3^S209F^ and AD, evidence for its impact on in vivo AD biomarkers is limited. Notably, Olive et al. [35] found no significant association between the variant and CSF Aβ42 or Aβ PET signal in cognitively normal individuals.

APOEε4 does not only influence Aβ accumulation but also directly modulates microglial function [28, 38, 47]. The APOEε4 isoform has been shown to alter microglial responses by limiting their ability to adopt a neuroprotective phenotype, impairing Aβ clearance and promoting their pro-inflammatory state [32, 33, 54]. The dual role of APOEε4 in Aβ pathology and immune regulation makes it a particularly relevant modifier when exploring microglial activity and gene–gene interactions in AD.

Given that both APOEε4 and ABI3 influence microglial function, albeit through distinct molecular pathways, their combined analysis may provide a more comprehensive understanding of microglial contributions to AD pathophysiology. Investigating these genes in parallel allows for the exploration of potential gene–gene interactions, additive effects, or pathway convergence that may influence cortical Aβ deposition and neuroinflammation. Also, due to the genetic and pathological heterogeneity of AD, replication of risk variants in distinct populations is essential to validate their generalizability and potential clinical relevance [3]. Studying the ABI3^S209F^ in a Finnish cohort provides an opportunity to confirm its association with AD risk in a genetically and environmentally distinct population, thereby strengthening the evidence for its role in disease pathogenesis [41, 52].

In this study, we aim to confirm the association of ABI3^S209F^ with AD in the Finnish FinnGen cohort, and to investigate how this variant, alone or in combination with APOEε4, modulates cortical Aβ burden, neuroinflammation, and brain morphology in cognitively healthy older adults using positron emission tomography (PET) and magnetic resonance imaging (MRI).

## Materials and methods

### Study population

The FinnGen Study is a large biobank-scale project that combines genome data and longitudinal register-based healthcare data of > 500,000 Finns [29]. In this study we used data from FinnGen release R12, which included 8,490 heterozygous carriers of ABI3^S209F^ and 511,670 non-carriers. For survival analysis, each ABI3^S209F^ carrier was assigned with up to five non-carrier controls matched for sex and the year of birth.

For the imaging part of this study, we recruited 58 subjects of ≥ 50 years of age, based on power calculations on previous studies. The participants of this cross-sectional study were recruited in collaboration with the local Auria biobank and from the Finnish Geriatric Intervention Study to Prevent Cognitive Impairment and Disability (FINGER) [27] cohort. Genotype data was available for a subset of the biobank cohort through FinnGen study, allowing the biobank to directly contact persons with the ABI3^S209F^ and *APOE* ε4/ε3 or *APOE* ε3/ε3 genotype who had previously signed a biobank consent and an additional informed consent allowing the biobank to contact them if they are suitable for participating in a research study. Main exclusion criteria were dementia or cognitive impairment, any degenerative neurological disease, chronic inflammatory condition, and contraindication for MRI or PET imaging. Our study involved three distinct groups for comparative analysis (*n* = 19 + 19 + 20): 1) individuals possessing both the ABI3^S209F^ variant and the *APOE* ε4/ε3 genotype (ABI3^S209F^/ε4), 2) those with the ABI3^S209F^ variant and the *APOE* ε3/ε3 genotype (ABI3^S209F^/ε3), and 3) a non-carrier group with the major ABI3^S209F^ allele and *APOE* ε3/ε3 genotype (NC). The sample size for the imaging part of this study was based on a power analysis calculated from previous research results obtained with the same radiotracers [1, 14]. The assumption was that the study would have 90% power (1–β = 0.9, α = 0.05) to detect, depending on the tracer, a 10–20% difference in regional binding ratios between the ABI3^S209F^/ε4 and NC groups. The final sample size also aimed to account for potential dropouts. The ABI3^S209F^/ε3 group was assumed to show an effect, with results expected to fall between those of the primary comparison groups. The study was conducted in accordance with the Declaration of Helsinki, and the study was approved by the Ethical Committee of the Hospital District of Southwest Finland (Ref.No. 21/1801/2019). All participants signed written informed consent.

### Genotyping

ABI3^S209F^ is encoded by a single nucleotide substitution NM_016428.3:c.626 T > C (rs616338). Although the T allele (encoding phenylalanine) is the reference allele, it is the rare allele in this locus. Thus, we refer to the common C allele (encoding serine) as the major allele and the rare T allele as the minor (effect) allele, consistent with previous reports as well as allele frequency and evolutionary conservation of the serine residue across multiple species [41].

The FinnGen cohort has been genotyped with Illumina (Illumina Inc., San Diego, USA) and Affymetrix (Thermo Fisher Scientific, Santa Clara, CA, USA) chip arrays as part of the FinnGen Study. Chip genotype data were imputed using the Finnish population-specific imputation reference panel Sequencing Initiative Suomi project (SISu v4.2, Institute for Molecular Medicine Finland, University of Helsinki, Finland, http://sisuproject.fi). Majority of the FinnGen cohort (92%) had been directly genotyped for rs616338, but for a broader coverage, imputed genotypes were used in all analyses. Imputation INFO score for rs616338 was 0.995. FINGER cohort [27] was genotyped with Illumina Infinium Global Screening Array and imputed with TOPMed reference panel as described before [3]. To confirm the *ABI3* genotypes in a subset of 42 individuals enrolled to the imaging study, venous blood was collected and genomic DNA was extracted from whole blood using QIAamp DNA Blood Mini Kit (Qiagen, Hilden, Germany). *ABI3* rs616338 was genotyped with TaqMan assay (C_2270073_20, Applied Biosystems). All genotypes were consistent with those observed with chip genotyping or imputation.

### Definition of disease endpoints

To define the disease endpoints, we utilized FinnGen core endpoints, which are based on digital health record data from Finnish health registries. Diagnoses are based on International Classification of Diseases (ICD) codes and have been harmonized over ICD-8, ICD-9 and ICD-10 codes.

AD was defined as a diagnosis in the hospital discharge or cause of death registries with the ICD codes G30 (ICD-10) or 3310 (ICD-9). AD onset age for the survival analysis was defined as the age of the first diagnosis. The remaining individuals were considered as controls. For the survival analysis, end of follow-up for controls was defined as death, moving abroad, or the latest update of the registry data, whichever came first.

Neurodegenerative disorder was defined as at least three entries with the following criteria: diagnosis in the hospital discharge or cause of death registries with the ICD codes F00*, G30 (ICD-10), 3310 (ICD-9), or 29010 (ICD-8); Finnish Social Insurance Institution (Kela) reimbursement for F00* or G30 (ICD-10) or reimbursement code 307; or prescription medicine purchases with ATC class N06D. Remaining individuals, excluding cases with AD, were considered as controls.

Dementia was defined as at least three entries with the following criteria: a diagnosis in the hospital discharge or cause of death registries with the ICD codes F00-F09 (ICD-10), 290, 3310, or 4378 A (ICD-9) or 290 (ICD-8), Finnish Social Insurance Institution (Kela) reimbursement code 307, or prescription medicine purchases with ATC class N06D. Dementia cases included individuals with vascular dementia, dementia in other diseases classified elsewhere, or unspecified dementia. Remaining individuals, excluding cases with organic mental disorders, were considered as controls.

### Brain imaging

All subjects underwent a structural brain MRI including T1-weighted sequences. Structural brain images were acquired with the Philips Ingenuity 3.0 T TF PET/MRI (Philips Healthcare, Amsterdam, the Netherlands). MRI was used to acquire volumetric variables for hippocampus, parahippocampus, entorhinal cortex and amygdala.

PET scans were acquired using an ECAT high-resolution research tomograph (HRRT, Siemens Medical solutions, Knoxville, TN). To estimate brain Aβ accumulation, [^11^C]PiB scans (*n* = 56) were acquired 40 to 90 min post injection (mean injected dose 493 (standard deviation (SD) 42) MBq). To estimate microglial activity, we used TSPO imaging with [^11^C]PK11195. Dynamic [^11^C]PK11195 scans (*n* = 56) were acquired for 60 min post injection (mean injected dose 476 (SD 41) MBq). All images were reconstructed with 3D ordinary Poisson ordered subset expectation maximization algorithm (OP-OSEM3D), and list mode data was histogrammed into 8 (6 × 5 + 2 × 10 min, [^11^C]PiB) and 17 (2 × 15; 3 × 30; 3 × 60; 7 × 300; 2 × 600 s, [^11^C]PK11195) time frames.

### Cognitive testing

A thorough neurocognitive test battery was administered to the participants by trained psychology students. The test battery included parts from the Finnish version of the Wechsler Memory Scale (WMS-R) and the Wechsler Adult Intelligence Scale (WAIS-R), Boston Naming Test (BNT), Trail Making Tests A and B (TMT-A and TMT-B), S-fluency, categorical fluency, and Stroop [2, 23, 43, 50, 51]. Domain-specific neurocognitive test z-scores, based on an a priori hypothesis [31], were calculated for executive functions, processing speed, language, and episodic memory, with higher scores indicating better performance. The executive function domain included the Trail Making Test A and B (TMT-B minus TMT-A), Stroop test (inhibition minus naming), digit span backward, and S-fluency. The processing speed domain consisted of TMT-A and digit symbol tests. The episodic memory domain included the WMS-R delayed logic memory and delayed verbal recall. The language domain included categorical fluency, the Boston naming test, and WAIS-R similarities.

### Brain image analysis

Both PET and MRI images were analysed for region of interest (ROI) and voxel-wise differences between the study groups. PET and MRI image preprocessing and analysis were performed using an automated pipeline at Turku PET Centre [26], which executed the PET data frame by frame realignment, PET-MRI co-registration, FreeSurfer (Freesurfer v6, https://surfer.nmr.mgh.harvard.edu/) ROI parcellation and PET data kinetic modelling. Regional and voxel level [^11^C]PiB binding was quantified as standardized uptake value ratios (SUVR) calculated for 60 to 90 min post injection using the cerebellar cortex as the reference region. A composite neocortical [^11^C]PiB score was calculated as the volume weighted average of the [^11^C]PiB region-to-cerebellar cortex SUVRs for the lateral frontal, lateral temporal, and parietal cortices as well as the posterior cingulate, anterior cingulate, and precuneus. This composite [^11^C]PiB score was used to estimate brain Aβ load. [^11^C]PK11195-binding was quantified from the same composite region, as distribution volume ratios (DVR) within 20–60 min post injection using a reference tissue input Logan’s method with pseudo-reference region extracted using supervised clustering algorithm [46, 53]*.* Voxel-level kinetic modeling for [^11^C]PK11195 was carried out using basis function implementation of simplified reference tissue model with respect to the aforementioned clustered pseudo-reference region and with 300 basis functions calculated within the Θ3 parameter limits 0.06 ≤ Θ3 ≤ 0.6 [19]*.* Partial volume effect (PVE)-corrected data was used for all [^11^C]PK11195 analysis to minimize the effect of PK binding in sinuses to cortical regions. PVE correction was carried out using PETPVE12 toolbox [17] in both ROI (geometric transfer matrix method) and voxel-level (Muller-Gartner method) data.

The brain MRI images were also analysed for volumetric differences extracted from the FreeSurfer results. Here we focused on the hippocampus, parahippocampus, and the entorhinal cortex due to their known association with neurodegeneration related to AD [7]. All voxel-wise analyses were conducted using statistical parametric mapping (SPM12 v12; Wellcome Trust Centre for Neuroimaging, London, UK) running on MATLAB R2021b (Math-Works, Natick, MA, USA).

### Statistical analysis

Association of rs616338-T allele with AD, neurodegenerative disorder, and dementia were assessed in FinnGen data with chi-square test using R 4.3.2. The data are presented as OR with 95% confidence intervals (CI). Survival analysis comparing AD-free survival of ABI3^S209F^ carriers and age- and sex-matched controls was performed in R 4.3.2 where Kaplan–Meier curves were generated using package survminer v0.4.9 and cox proportional hazards models were fitted with survival package v3.2–7 [44, 45]. The proportional hazards assumption was assessed using the Schoenfeld residuals test, which indicated a violation for the *APOE* genotype. To account for this, the Cox model was stratified by *APOE* genotype. The data is presented as hazard ratio (HR) with 95% CI. Results with *p* < 0.05 were considered statistically significant.

Statistical analyses of the imaging data were performed using JMP Pro 16.0.0 (SAS Institute Inc., Cary, North Carolina, USA). All data following a normal distribution are presented as mean (SD), otherwise as median (interquartile range, IQR). The normality of the data was evaluated visually from the distribution and with the Shapiro–Wilk test. Differences in continuous variables between the three groups were tested using linear regression models, adjusting for age and sex. The MRI variables were also adjusted for total intracranial volume. If a significant effect was found, all pairs were compared using the post hoc Tukey’s honest significance test for multiple comparisons.

Volumetric differences in T1 MRI, [^11^C]PIB, and [^11^C]PK11195-binding at the voxel level were assessed using ANCOVA, using age and sex as covariates for [^11^C]PIB and [^11^C]PK11195, but also total intracranial volume for MRI. This was followed by post-hoc pairwise comparisons in SPM12. Voxel-wise differences between the groups were tested using linear regression in SPM12 to evaluate if differences were present also outside the a priori chosen brain regions. Uncorrected *p* < 0.001 combined with a cluster-level false discovery rate (FDR) correction for multiple comparisons was considered statistically significant in the voxel-based analyses. When significant FDR corrected clusters were found we applied family wise error (FWE) correction with *p* < 0.05 to see if the results survived the tighter threshold.

To combine the scores of different neurocognitive tests into domain-specific scores, z-scores for the tests were calculated by standardizing the raw test scores to the study population’s mean and standard deviation. To achieve a normal distribution, one outlier per raw test were excluded before the z-transformation (1 outlier in Boston naming test; and 1 outlier in Stroop test). All outliers performed worse on the cognitive tests than –2 SD of the present study population. A skewness of –1 to 1 was accepted for the raw test score distribution. Domain-specific z-scores were determined by averaging all the z-scores within each domain. For tests with a reverse scale (Stroop, TMT-A, and TMT-B), reciprocal numbers were used to ensure higher scores indicated better performance. Participants with missing test results were excluded.

## Results

### ABI3^S209F^ associates with increased risk and earlier onset age of AD in FinnGen cohort

**Fig. 1:**
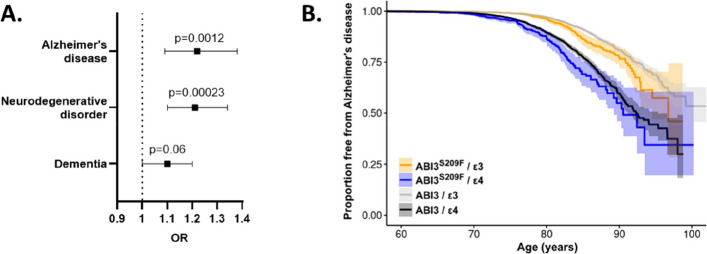
ABI3^S209F^ associates with increased risk and earlier onset age of AD in FinnGen. **A** Forest plot showing ABI3^S209F^ association with selected disease endpoints in FinnGen cohort (ABI3^S209F^ *n* = 8,490; ABI3 *n* = 511,670). Odds ratio (OR) with 95% confidence intervals. **B** Kaplan–Meier survival curves of time to AD diagnosis in different ABI3 and APOE genotype groups in FinnGen cohort. Shading indicates 95% confidence intervals. X-axis indicates age at the first diagnosis for cases and age at the end of follow-up for controls. N(ABI3^S209F^/ε3) = 5141, N(ABI3^S209F^/ε4) = 2326, N(ABI3/ε3) = 47,234, N(ABI3^S209F^/ε4) = 21,484

ABI3^S209F^ (rs616338-T) was significantly associated with increased risk of AD (OR = 1.22, 95% CI: 1.09–1.38, *p* = 0.0012) and neurodegenerative disorder (OR = 1.21, 95% CI: 1.10–1.34, *p* = 0.00023) in FinnGen (Fig. 1A). Association with dementia was not statistically significant (OR = 1.10, 95% CI: 1.00–1.20, *p* = 0.06).

To investigate the impact of ABI3^S209F^ and *APOE* ε4 on the age of onset of AD, we performed survival analysis for age at AD diagnosis. Kaplan–Meier survival curves indicated an earlier onset of AD among the ABI3^S209F^ carriers when compared to the non-carriers with the same *APOE* genotype (Fig. 1B). Stratified Cox proportional hazards models were used to account for the violation of the proportional hazard assumption by *APOE* genotype. Within the *APOE* ε3 stratum, ABI3^S209F^ carriers exhibited a significantly increased hazard of AD onset (HR = 1.31, 95% CI: 1.09–1.59, *p* = 0.0046). Similarly, in the *APOE* ε4 stratum, ABI3^S209F^ carriers showed an elevated risk (HR = 1.32, 95% CI: 1.09–1.60, *p* = 0.0045). These findings suggest that the ABI3^S209F^ variant is associated with earlier AD onset, independent of *APOE* genotype.

### Demographics of the PET–MRI subjects

Baseline demographic, clinical, and imaging characteristics of the PET–MRI subsample are presented in Table 1. Across the three genotype-defined study groups (ABI3^S209F^/ε4, ABI3^S209F^/ε3, and NC), no statistically significant differences were observed in sex distribution, age, height, or hippocampal volume (P > 0.07 for all). Cognitive performance, as assessed by MMSE, was similar across groups. Importantly, a significant group difference was observed in cortical Aβ burden, estimated as composite [11C]PiB SUVR, with the ABI3^S209F^/ε3 group showing a lower median binding score than the ABI3^S209F^/ε4 group (*p* = 0.01). No differences were observed in microglial activation ([11C]PK11195 DVR).

**Table 1:** Subject demographics and descriptive data

		***ABI3***^***S209F***^***/ε4***	***ABI3***^***S209F***^***/ε3***	***NC***	**Group difference**
N		19	19	20	
Sex	female	11	8	11	0.58
	male	8	11	9	
Age	Mean (sd)	69.5 (6.2)	71.4 (6.9)	71.4 (5.1)	0.55
Height	Mean (sd)	168.9 (9.4)	172.2 (9.6)	168.3 (7.9)	0.39
Weight	Mean (sd)	81.8 (12.7)	83.4 (18)	73.1 (13.2)	0.073
BMI	Mean (sd)	28.8 (4.5)	27.9 (4.2)	25.7 (3.7)	0.066
[^11^C]PiB score	Mean (sd))	2.0 (0.7)	1.67 (0.5)*	1.75 (0.6)	**0.01**
[^11^C]PK DVR	Mean (sd)	1.3 (0.1)	1.3 (0.1)	1.3 (0.1)	0.83
Hippocampal volume	Mean (sd)	7.7 (0.8)	7.7 (1.0)	7.2 (0.6)	0.26
MMSE	Median (IQR)	28.5 (28–29)	29 (28–29)	29 (28–30)	0.83

Data are presented as mean (standard deviation) or median (interquartile range) depending on the distribution. Differences between groups were tested with ANCOVA with Tukey’s honest significance test, Kruskal–Wallis test with Dunn’s method for multiple or linear models. χ2 test was used for testing categorical variables. *P* value presents overall difference between groups. Significant differences in pairwise comparisons to ABI3^S209F^/e4e3 (*p* < 0.05 = *) are presented

### APOEε4, but not ABI3^S209F^, increases brain Aβ load

**Fig. 2:**
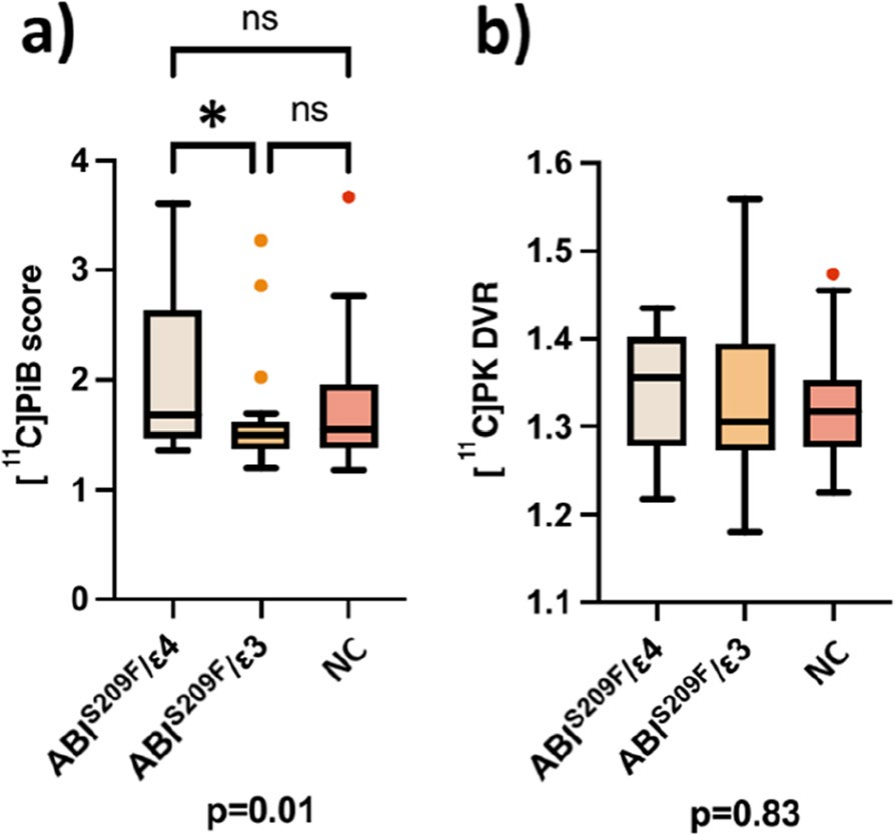
**a** [^11^C]PiB score and **b** [^11^C]PK11195 DVR stratified by the three study groups. Median, first and third quartile and range are presented by the box plot. * = *p* = 0.017 (Tukey´s honest significance test). *P*-value below the figures presents overall difference between the three groups (ANCOVA)

**Fig. 3:**
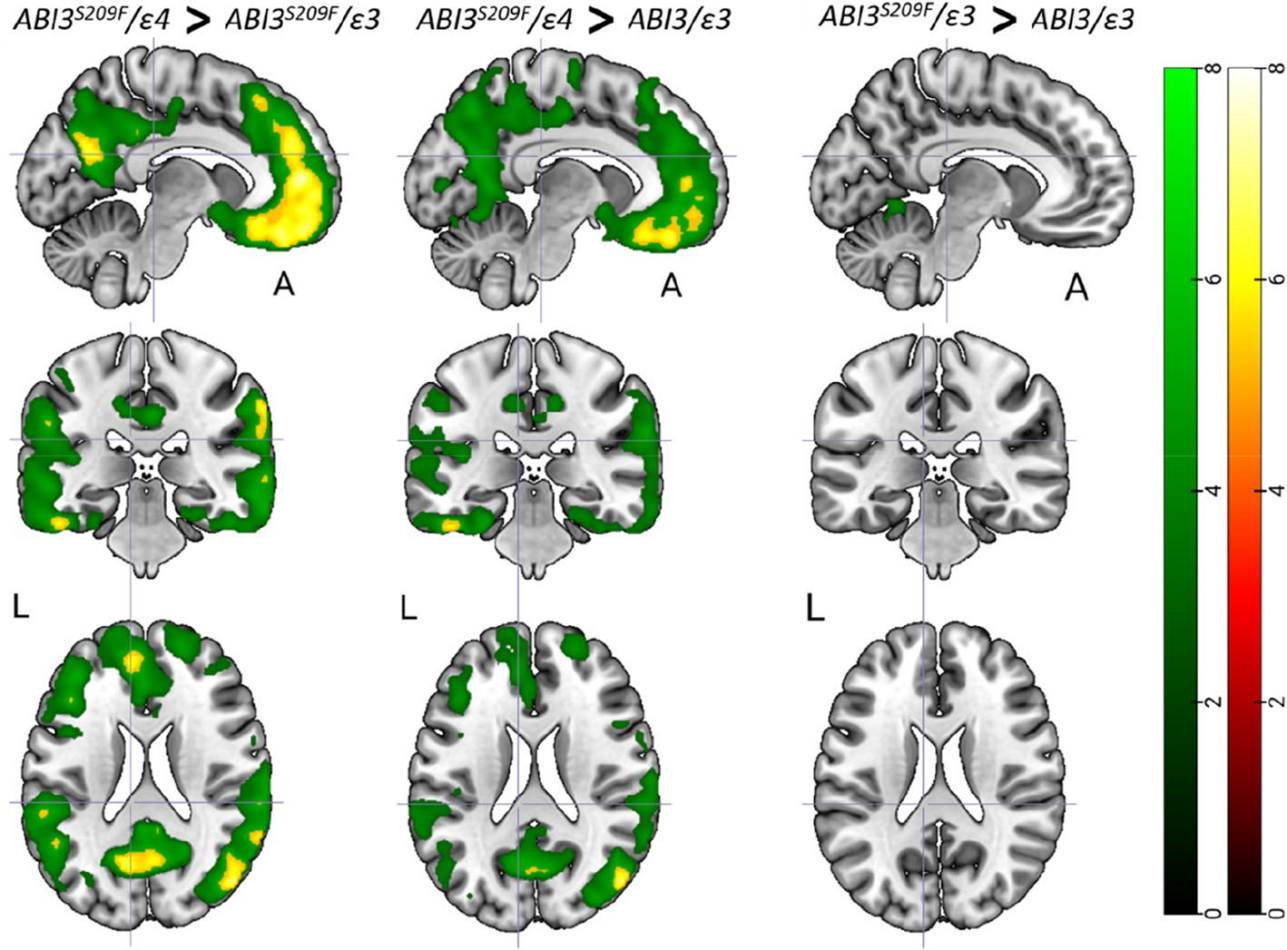
Voxel-wise analysis showing areas with statistically significant increase in [^11^C]PiB score between the ABI3^S209F^/ε4, ABI3^S209F^/ε3 and NC groups. FDR corrected *p* < 0.001 in green colour. FWE corrected *p* < 0.05 in red-yellow

In the ROI analysis, the ABI3^S209F^/ε4 had higher composite cortical [^11^C]PiB score when compared to the ABI3^S209F^/ε3 (2.0 (0.7) vs. 1.67 (0.5); mean (sd), *p* = 0.017 (HST)) (Fig. 2a). There was no difference between the ABI3^S209F^/ε3 and NC (1.67 (0.5) vs 1.75 (0.6), *p* = 0.75 (HST)) or ABI3^S209F^/ε4 and NC (2.0 (0.7) vs. 1.75 (0.6), *p* = 0.76 (HST)). The voxel-level analysis showed higher [^11^C]PiB binding across the cortex in the ABI3^S209F^/ε4 group than in the two other groups (Fig. 3).

### No global, but subtle regional neuroinflammatory differences in ABI3^S209F^/ε4 carriers

**Fig. 4:**
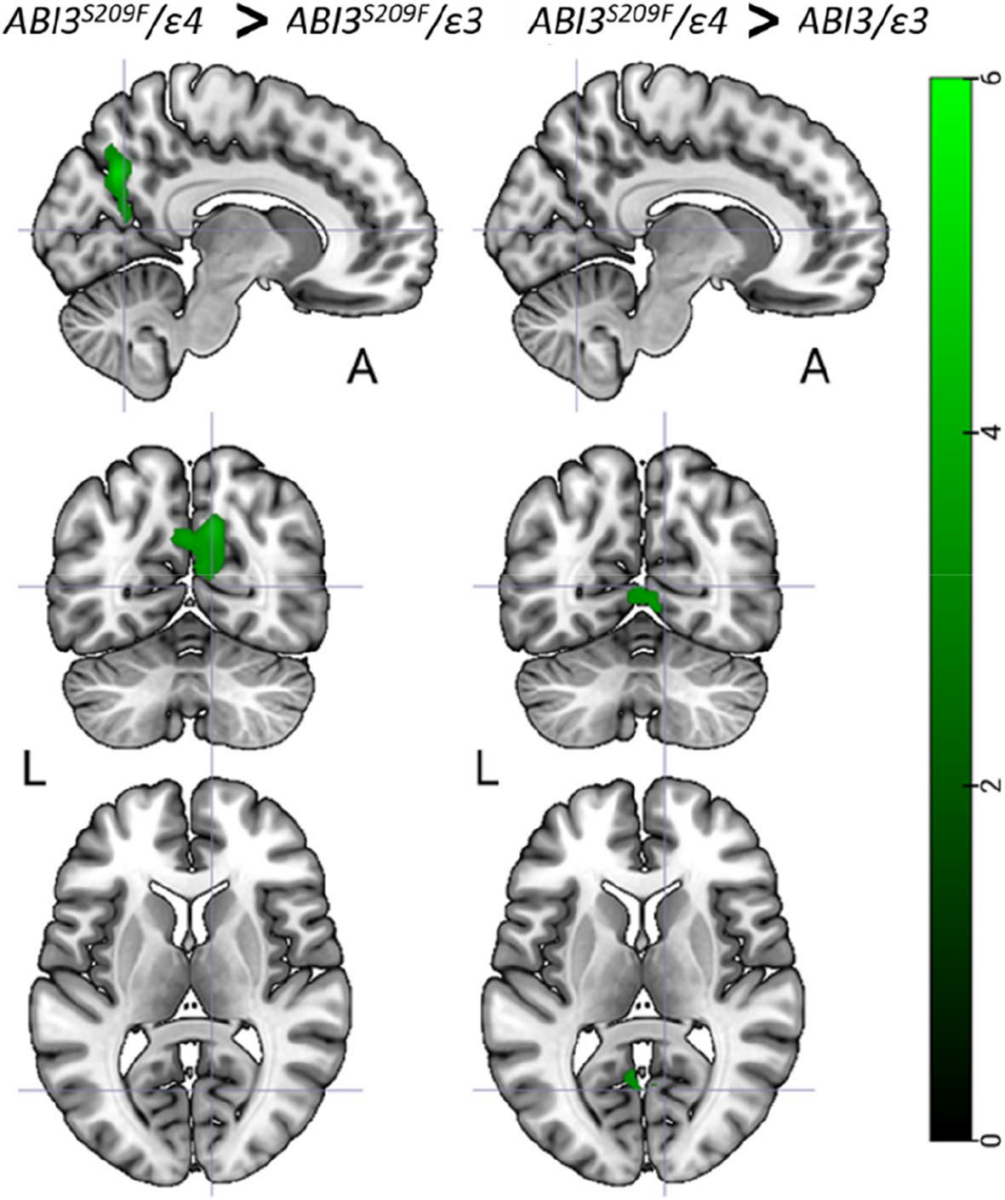
ABI3^S209F^/ε4 group had higher [^11^C]PK11195 binding than the ABI3^S209F^/ε3 or NC groups. FDR corrected *p* < 0.001 in green colour

The [^11^C]PK11195 analysis revealed no significant differences between the groups in the composite cortical ROI (Fig. 2b). However, voxel-level analysis detected minor differences between the ABI3^S209F^/ε4 and both the ABI3^S209F^/ε3 and NC groups, but not between the ABI3^S209F^/ε3 and NC groups (Fig. 4). The ABI3^S209F^/ε4 showed higher [^11^C]PK11195 binding in the precuneus and parieto-occipital regions compared to the ABI3^S209F^/ε3 group. Additionally, higher [^11^C]PK11195 binding was observed in the parieto-occipital and calcarine regions of the ABI3^S209F^/ε4 compared to the NC group.

While no significant ROI-level correlations were found between the PET tracers, some voxel-wise associations were identified mainly in subcortical regions in the white matter. In addition, a weak correlation (*r* = 0.27, *p* = 0.043) was observed between [11C]PK11195 binding and cortical volume. Full correlation results are provided in the Supplementary Materials (Table S1, Figure S1).

### ABI3^S209F^ does not significantly affect brain region volumes

In the prechosen ROIs we found no differences between the groups in the volumes of cerebral cortex, hippocampus, parahippocampus, entorhinal cortex or amygdala (Supplementary material, Table S2). Subsequent VBM analysis found that the *ABI3*^*S209F*^/ε3 had lower volumes than the other groups mainly in the superior temporal gyrus, when correcting for FDR, but these findings did not survive the FEW correction (Supplementary material, Figure S2).

### ABI3^S209F^ has no effect on cognition

**Fig. 5:**
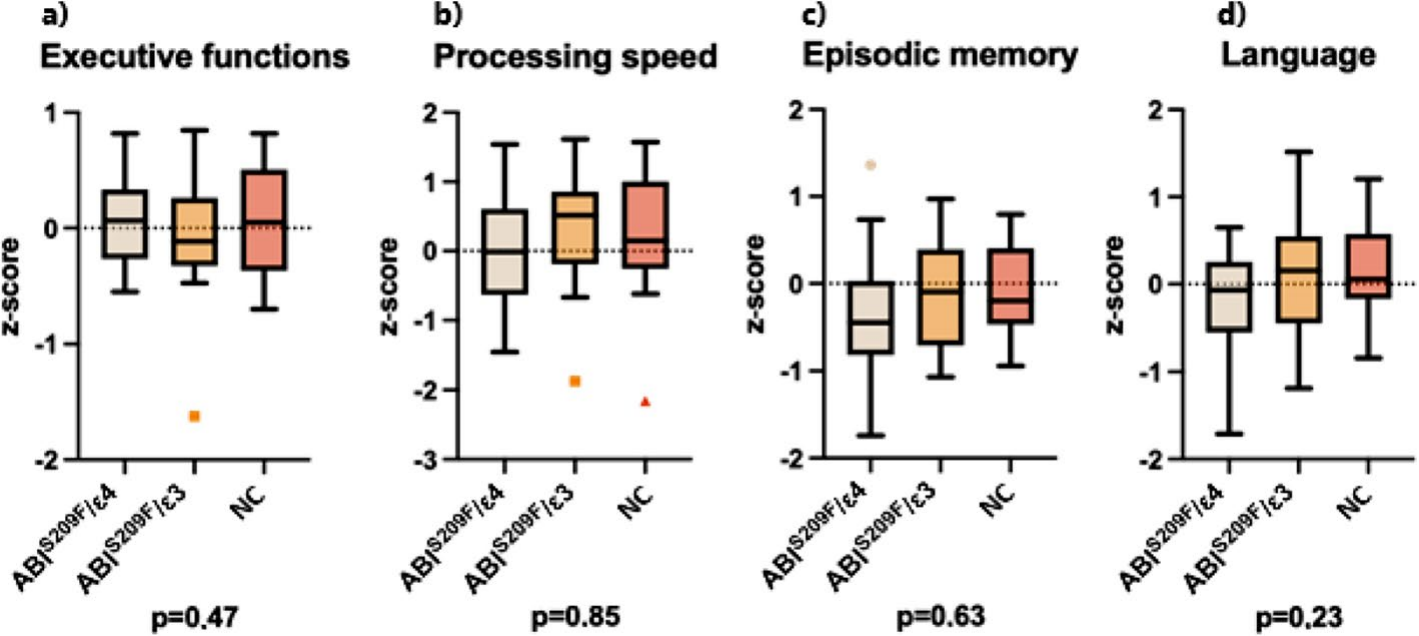
Domain-specific cognitive performance. Box plots represent z-standardized composite scores for **a** Executive functions, **b** Processing speed, **c** Episodic memory, and **d** Language. *P*-value below the figures presents overall difference between the three groups (ANCOVA)

Global cognition was assessed with the MMSE, and domain-specific performance was summarized as z-standardized composite. One-way ANOVA followed by Tukey’s honest significant difference test revealed no overall between-group differences for MMSE (Table 1) or any cognitive domain (Table 2, Fig. 5).

**Table 2:** Cognitive test results

		***ABI3***^***S209F***^***/ε4***	***ABI3***^***S209F***^***/ε3***	***NC***	**Group difference**
Executive functions	Mean (sd)	0.041 (0.41)	−0.046 (0.61)	0.035 (0.47)	0.47
Processing speed	Mean (sd)	0.024 (0.79)	0.30 (0.86)	0.21 (0.87)	0.85
Episodic memory	Mean (sd)	−0.34 (0.73)	−0.11 (0.65)	−0.10 (0.52)	0.63
Language functions	Mean (sd)	−0.16 (0.58)	0.12 (0.66)	0.14 (0.53)	0.23

Data are presented as mean (standard deviation). Differences between groups were tested with one-way ANOVA with Tukey’s honest significance test. *P* value presents overall difference between groups. Significant differences in pairwise comparisons to ABI ^S209F^/e4e3 (*p* < 0.05 = *) are presented

## Discussion

This study investigated the ABI3^S209F^ genetic variant as a risk factor for AD using data from the large Finnish FinnGen cohort. In addition, we experimentally assessed its impact, alone or combined with the APOEε4, on cortical Aβ deposition, neuroinflammation, brain morphology, and cognitive performance in a separate subsample of cognitively healthy older adults.

Genetic analysis confirmed ABI3^S209F^ as a significant AD risk variant (OR = 1.22), though with a somewhat smaller effect size compared to previous findings [8, 10, 35, 39, 41, 52]. Additionally, survival analyses indicated that ABI3^S209F^ carriers developed AD earlier than non-carriers. In the experimental analyses, ABI3^S209F^ alone did not influence cortical Aβ accumulation, neuroinflammation or regional brain volumes. However, subtle regional effects emerged in voxel-wise analyses, particularly for ABI3^S209F^ combined with APOEε4, suggesting region-specific Aβ deposition and modest neuroinflammation not detected by broader ROI analyses. Collectively, these findings replicate and extend prior results, highlighting the complexity and context-dependent nature of ABI3^S209F^’s contribution to AD pathology.

In a genetic association analysis in FinnGen, the ABI3^S209F^ variant significantly associated with AD and more broadly defined neurodegenerative disorder, with comparable ORs across the endpoints. Interestingly, no significant association was observed with the broadest endpoint definition, dementia. This likely reflects differences in definitions of the broader endpoints: while the neurodegenerative disorder endpoint may more specifically capture AD and related pathologies, the dementia endpoint encompasses a heterogeneous group of conditions, including vascular and unspecified dementias. The lack of association with dementia may therefore result from dilution of AD-specific genetic signals within this phenotypically diverse group.

Beyond AD risk, ABI3^S209F^ also appears to influence the timing of disease onset. Survival analysis revealed that carriers of the variant developed AD at an earlier age compared to non-carriers, with consistent effect observed across *APOE* ε3/ε3 and ε3/ε4 backgrounds. These findings suggest that ABI3^S209F^ may act as a disease modifier, accelerating the onset of AD independently of *APOE* genotype. Taken together, these results reinforce the specificity of the association of ABI3^S209F^ variant with AD and highlight its potential role in modulating disease progression.

In the imaging part of the study, we examined the effect of ABI3^S209F^, in the presence or absence of the APOEε4, on brain Aβ deposition, neuroinflammation, brain structures, and cognitive functions in cognitively healthy older adults. Although ABI3^S209F^ has previously been associated with increased AD risk in several studies, including replication in our current study, we did not observe increased cortical Aβ deposition in ABI3^S209F^/ε3 carriers when compared to NC, either in ROI-based or voxel-wise analyses. This suggests that ABI3^S209F^ alone, without the *APOE* ε4 allele, may have limited functional impact on Aβ accumulation in cognitively normal individuals.

Interestingly, the highest cortical Aβ levels in ROI analysis were observed in participants carrying both ABI3^S209F^ and APOEε4. However, these were only significantly different compared to the ABI3^S209F^/ε3 group, not to NC. While group sizes were balanced, this lack of significance relative to the NC group likely reflects a lack of statistical power to detect minute, global Aβ increases in a cognitively healthy cohort where pathology is still in its infancy. Voxel-wise analysis, in contrast, revealed region-specific increases in [^11^C]PiB signal in the ABI3^S209F^/ε4 group compared to both other groups, particularly in frontal, temporal, and posterior cortices. Previous work has shown that one APOEε4 alone does not consistently induce such voxel-level differences [37, 42] supporting a gene–gene interaction hypothesis. While our ROI findings align with previous reports showing no significant global association between ABI3^S209F^ and Aβ markers in cognitively healthy individuals [35], the voxel-level differences suggest that this genetic combination leads to localized Aβ accumulation that conventional ROI analyses fail to capture. Animal studies have yielded mixed results regarding ABI3’s role in Aβ pathology. While ABI3 knockout has been associated with increased Aβ deposition in some studies [24], others have reported reduced deposition, particularly at early time points [20]. In the latter case, the reduction was transient, and Aβ levels eventually caught up with NC as the animals aged. While APOE ε4 remains the single greatest genetic modulator of sporadic AD risk [9], recent research on ABI3^S209F^ reveals that both factors drive disease progression by inducing microglial dysfunction [8, 12, 21, 41]. In microglia APOE ε4 is known to affect upregulation of inflammatory and immune-related genes, produce higher levels of pro-inflammatory cytokines, impairs the ability of microglia to phagocytose and clear Aβ plaques, downregulation of receptors like P2RY12 and TREM2 which are essential for microglial chemotaxis and phagocytosis [12, 16].

Parallel to this, studies with ABI3 knock-out models have demonstrated severely impaired migration and phagocytosis [24, 25]. Altogether, these findings suggest a potent interaction where the presence of both ABI3^S209F^ and APOE ε4 likely creates a compound defect where microglial recruitment and clearance are simultaneously compromised.

To our knowledge, this is the first study to examine TSPO PET imaging in cognitively normal individuals carrying the ABI3^S209F^ variant. Previous studies with various PET-ligands have reported increased TSPO binding between AD and NC [4, 13, 15], and also in Aβ-positive MCI [34, 36] and Aβ-positive NC [14, 56]. However, when comparing a composite ROI covering the entire cortical grey matter, we found no significant differences in [^11^C]PK11195 binding between the genotype groups, although Aβ accumulation was clearly highest in the ABI3^S209F^/ε4 group in the same region. Voxel-wise analysis revealed a subtle increase in [^11^C]PK11195 binding in the parieto-occipital cortex of the ABI3^S209F^/ε4 group compared to the other groups. Although this region also showed elevated Aβ signal, the highest Aβ accumulation was observed in the frontal cortex, where no corresponding increase in [11C]PK11195 binding was detected. Our findings suggest that while the combination of ABI3^S209F^ and APOEε4 is associated with increased cortical Aβ accumulation, this does not correspond to a parallel increase in microglial activation as measured by [11C]PK11195. A similar dissociation has previously been reported among APOEε4 carriers with varying allele loads [42]. This mismatch may reflect the limited sensitivity of first-generation TSPO tracers such as [11C]PK11195, which are known to suffer from poor signal-to-noise ratio and high nonspecific binding [49, 55] or a temporal lag between Aβ deposition and immune response, or the functional heterogeneity of microglial activation, which is not fully captured by TSPO PET imaging [14]. Overall, while the voxel- and ROI-level data diverge in some respects, both support a nuanced and genotype-dependent role for ABI3^S209F^ in AD pathology.

The strength of this study is the combination of population-scale genetic data with in vivo multimodal imaging, enabling a rare genotype-specific characterization of early AD-related pathology in cognitively normal individuals. The use of the large and well-characterized FinnGen cohort allowed us to validate the ABI3^S209F^ AD association in a genetically distinct Northern European population, adding valuable replication evidence to earlier findings. A major practical strength of this study was the ability to efficiently recruit participants with specific genotypes through biobank collaboration. Without access to genotype-based preselection, recruiting a sufficient number of ABI3^S209F^ carriers would have required genotyping and screening several hundred individuals. The biobank-based recruitment thus greatly increased feasibility and resource efficiency. Additionally, the integration of PET imaging for both Aβ and microglial activity, alongside MRI-derived structural metrics and neuropsychological testing, enabled a multidimensional assessment of functional consequences at an early disease stage.

However, some limitations should be noted. The use of the first-generation TSPO tracer [11C]PK11195 may have constrained sensitivity to microglial activation due to its limited signal-to-noise ratio and high nonspecific binding. Also, the sample size in our imaging part was modest. While it was based on power calculations from previous studies, those calculations were primarily derived from symptomatic patient groups compared to controls where pathologies are more pronounced. Consequently, our study may have been underpowered to detect the more subtle changes characteristic of a cognitively healthy cohort. This increases the risk of a Type II error, particularly in ROI-based analyses where early focal signals may be diluted by the averaging of non-affected voxels. Furthermore, although voxel-wise analysis increases spatial precision and proved more sensitive in identifying these localized differences, it also introduces multiple comparison challenges and increases the risk of Type I error, despite statistical correction. Additionally, the recruitment of healthy volunteers may introduce a selection bias toward higher educational or socioeconomic status. However, because all study groups were recruited using identical methods, this ‘healthy volunteer effect’ is consistent across cohorts and unlikely to account for the observed intergroup differences. Nonetheless, we acknowledge that this may limit the generalizability of the findings to a broader demographic. Finally, while the study design allowed for the evaluation of *ABI3*^S209F^ effects in isolation and in combination with *APOE* ε4, the cross-sectional nature of the imaging component precludes firm conclusions about temporal dynamics or causality.

Despite these limitations, the present findings highlight the importance of considering gene–gene interactions and regional brain vulnerability in understanding AD risk. Further studies using larger imaging cohorts, more sensitive second-generation tracers, and longitudinal follow-up will be essential to clarify the mechanisms by which ABI3 and APOE ε4 interact to influence early neuropathological changes.

## Conclusions

This study confirms the association of the ABI3^S209F^ variant with increased risk for AD. The variant was also associated with an earlier age of AD onset. ABI3^S209F^ alone does not significantly affect cortical Aβ deposition or neuroinflammation. However, the combination of ABI3^S209F^ and APOEε4 may contribute to region-specific Aβ accumulation and increased microglial activation. Our findings emphasize the complex and context-dependent role of ABI3^S209F^ in AD pathophysiology, possibly acting as a modifier rather than a primary driver of pathology. The lack of consistent differences in cognitive performance and brain volumes supports the notion that ABI3^S209F^-related changes may precede symptomatic disease or require additional genetic or environmental interactions to manifest. Further longitudinal and mechanistic studies are needed to elucidate the functional consequences of this variant and its potential as a therapeutic target or biomarker in preclinical stages of AD.

## Supplementary Information

Supplementary Material 1.

## Data Availability

The brain imaging datasets used and analysed during the current study are available from the corresponding author on reasonable request. Summary statistics from FinnGen data release 12 are publicly available and can be accessed at [https://www.finngen.fi/en/access_results](https:/www.finngen.fi/en/access_results) and [https://r12.finngen.fi](https:/r12.finngen.fi). Access for individual level genotype data can be applied for via the Fingenious portal ([https://site.fingenious.fi/en/](https:/site.fingenious.fi/en)) hosted by the Finnish Biobank Cooperative FinBB ([https://finbb.fi/en/](https:/finbb.fi/en)). Access to Finnish Health register data can be applied from Findata ([https://findata.fi/en/data/](https:/findata.fi/en/data)).

## Abbreviations

AD: Alzheimer’s disease
ApoE: Apolipoprotein E
APOEε4: APOE ε4 allelle
Aβ: Beta-amyloid
ABI3: Abelson interactor family member 3
ABI3^S209F^: ABI3 p.Ser209Phe variant
APP: Amyloid precursor protein
ATC: Anatomical Therapeutic Chemical
BNT: Boston Naming Test
CSF: Cerebrospinal fluid
DVR: Distribution Volume Ratio
FDR: False discovery rate
FINGER: Finnish Geriatric Intervention Study to Prevent Cognitive Impairment and Disability
FinnGen: Finnish genetic research project combining genomic and health registry data
FWE: Family-wise error
GWAS: Genome-wide association study
HRRT: High-resolution research tomograph
ICD: International Classification of Diseases
IQR: Interquartile range
MBq: Megabecquerel
MCI: Mild cognitive impairment
MRI: Magnetic resonance imaging
PET: Positron emission tomography
PVE: Partial volume effect
ROI: Region of interest
SD: Standard deviation
SPM: Statistical parametric mapping
SUVR: Standardized uptake value ratio
TSPO: Translocator protein

## References

[1] Aalto S, Scheinin NM, Kemppainen NM, Någren K, Kailajärvi M, Leinonen M, et al. Reproducibility of automated simplified voxel-based analysis of PET amyloid ligand [11C]PIB uptake using 30-min scanning data. Eur J Nucl Med Mol Imaging. 2009;36(10):1651–60. 10.1007/s00259-009-1174-1. 19495749 10.1007/s00259-009-1174-1

[2] Battery AIT. Manual of directions and scoring. Washington DC: War Department, Adjutant General’s Office; 1944.

[3] Bellenguez C, Küçükali F, Jansen IE, Kleineidam L, Moreno-Grau S, Amin N, et al. New insights into the genetic etiology of Alzheimer’s disease and related dementias. Nat Genet. 2022;54(4):412–36. 10.1038/s41588-022-01024-z. 35379992 10.1038/s41588-022-01024-z PMC9005347

[4] Cagnin A, Brooks DJ, Kennedy AM, Gunn RN, Myers R, Turkheimer FE, et al. In-vivo measurement of activated microglia in dementia. Lancet. 2001;358(9280):461–7. 10.1016/S0140-6736(01)05625-2. 11513911 10.1016/S0140-6736(01)05625-2

[5] Castellano JM, Kim J, Stewart FR, Jiang H, Demattos RB, Patterson BW, et al. Human apoE isoforms differentially regulate brain amyloid-β peptide clearance. Sci Transl Med. 2011;3(89):89ra57-89ra57. 10.1126/scitranslmed.3002156. 21715678 10.1126/scitranslmed.3002156 PMC3192364

[6] Castillo E, Leon J, Mazzei G, Abolhassani N, Haruyama N, Saito T, et al. Comparative profiling of cortical gene expression in Alzheimer’s disease patients and mouse models demonstrates a link between amyloidosis and neuroinflammation. Sci Rep. 2017;7(1). 10.1038/s41598-017-17999-3. 10.1038/s41598-017-17999-3 PMC5736730 29259249

[7] Chandra A, Dervenoulas G, Politis M. Magnetic resonance imaging in Alzheimer’s disease and mild cognitive impairment. J Neurol. 2019;266(6):1293–302. 10.1007/s00415-018-9016-3. 30120563 10.1007/s00415-018-9016-3 PMC6517561

[8] Conway OJ, Carrasquillo MM, Wang X, Bredenberg JM, Reddy JS, Strickland SL, et al. ABI3 and PLCG2 missense variants as risk factors for neurodegenerative diseases in Caucasians and African Americans. Mol Neurodegener. 2018;13(1). 10.1186/s13024-018-0289-x. 10.1186/s13024-018-0289-x PMC6190665 30326945

[9] Corder EH, Saunders AM, Strittmatter WJ, Schmechel DE, Gaskell PC, Small GW, et al. Gene dose of Apolipoprotein E type 4 allele and the risk of Alzheimer’s disease in late onset families. Science. 1993;261(5123):921–3. 10.1126/science.8346443. 8346443 10.1126/science.8346443

[10] Dalmasso MC, Brusco LI, Olivar N, Muchnik C, Hanses C, Milz, E, et al. Transethnic meta-analysis of rare coding variants in PLCG2, ABI3, and TREM2 supports their general contribution to Alzheimer’s disease. Transl Psychiatry. 2019;9(1). 10.1038/s41398-019-0394-9. 10.1038/s41398-019-0394-9 PMC6355764 30705288

[11] Davidson AJ, Ura S, Thomason PA, Kalna G, Insall RH. Abi is required for modulation and stability but not localization or activation of the SCAR/WAVE complex. Eukaryot Cell. 2013;12(11):1509–16. 10.1128/ec.00116-13. 24036345 10.1128/EC.00116-13 PMC3837927

[12] Dias D, Portugal CC, Relvas J, Socodato R. From genetics to neuroinflammation: the impact of ApoE4 on microglial function in Alzheimer’s disease. Cells. 2025;14(4):243. 39996715 10.3390/cells14040243 PMC11853365

[13] Edison P, Archer HA, Gerhard A, Hinz R, Pavese N, Turkheimer FE, et al. Microglia, amyloid, and cognition in Alzheimer’s disease: an [11C](R)PK11195-PET and [11C]PIB-PET study. Neurobiol Dis. 2008;32(3):412–9. 10.1016/j.nbd.2008.08.001. 18786637 10.1016/j.nbd.2008.08.001

[14] Fan Z, Brooks DJ, Okello A, Edison P. An early and late peak in microglial activation in Alzheimer’s disease trajectory. Brain. 2017;140(3):792–803. 10.1093/brain/aww349. 28122877 10.1093/brain/aww349 PMC5837520

[15] Fan Z, Okello AA, Brooks DJ, Edison P. Longitudinal influence of microglial activation and amyloid on neuronal function in Alzheimer’s disease. Brain. 2015;138(12):3685–98. 10.1093/brain/awv288. 26510952 10.1093/brain/awv288

[16] Fernandez CG, Hamby ME, Mcreynolds ML, Ray WJ. The role of APOE4 in disrupting the homeostatic functions of astrocytes and microglia in aging and Alzheimer’s disease. Front Aging Neurosci. 2019;11. 10.3389/fnagi.2019.00014. 10.3389/fnagi.2019.00014 PMC6378415 30804776

[17] Gonzalez-Escamilla G, Lange C, Teipel S, Buchert R, Grothe MJ. PETPVE12: an SPM toolbox for partial volume effects correction in brain PET – application to amyloid imaging with AV45-PET. Neuroimage. 2017;147:669–77. 10.1016/j.neuroimage.2016.12.077. 28039094 10.1016/j.neuroimage.2016.12.077

[18] Guerreiro RJ, Gustafson DR, Hardy J. The genetic architecture of Alzheimer’s disease: beyond APP, PSENs and APOE. Neurobiol Aging. 2012;33(3):437–56. 10.1016/j.neurobiolaging.2010.03.025. 20594621 10.1016/j.neurobiolaging.2010.03.025 PMC2980860

[19] Gunn RN, Lammertsma AA, Hume SP, Cunningham VJ. Parametric imaging of ligand-receptor binding in PET using a simplified reference region model. Neuroimage. 1997;6(4):279–87. 10.1006/nimg.1997.0303. 9417971 10.1006/nimg.1997.0303

[20] Ibanez KR, Mcfarland KN, Phillips J, Allen M, Lessard CB, Zobel L, et al. Deletion of Abi3/Gngt2 influences age-progressive amyloid β and tau pathologies in distinctive ways. Alzheimers Res Ther. 2022;14(1). 10.1186/s13195-022-01044-1. 10.1186/s13195-022-01044-1 PMC9327202 35897046

[21] Jackson RJ, Hyman BT, Serrano-Pozo A. Multifaceted roles of APOE in Alzheimer disease. Nat Rev Neurol. 2024;20(8):457–74. 10.1038/s41582-024-00988-2. 38906999 10.1038/s41582-024-00988-2 PMC12185264

[22] Jansen IE, Savage JE, Watanabe K, Bryois J, Williams DM, Steinberg S, et al. Genome-wide meta-analysis identifies new loci and functional pathways influencing Alzheimer’s disease risk. Nat Genet. 2019;51(3):404–13. 10.1038/s41588-018-0311-9. 30617256 10.1038/s41588-018-0311-9 PMC6836675

[23] Kaplan E, Goodglass H, Weintraub S. The Boston naming test. Philadelphia: Lea & Febiger; 1983.

[24] Karahan H, Smith DC, Kim B, Dabin LC, Al-Amin MM, Wijeratne HRS, et al. Deletion of ABI3 gene locus exacerbates neuropathological features of Alzheimer’s disease in a mouse model of Aβ amyloidosis. Sci Adv. 2021;7(45). 10.1126/sciadv.abe3954. 10.1126/sciadv.abe3954 PMC8565913 34731000

[25] Karahan H, Smith DC, Kim B, Mccord B, Mantor J, John SK, et al. The effect of Abi3 locus deletion on the progression of Alzheimer’s disease-related pathologies. Front Immunol. 2023;14. 10.3389/fimmu.2023.1102530. 10.3389/fimmu.2023.1102530 PMC9988916 36895556

[26] Karjalainen T, Tuisku J, Santavirta S, Kantonen T, Bucci M, Tuominen L, et al. Magia: robust automated image processing and kinetic modeling toolbox for PET neuroinformatics. Front Neuroinform. 2020;14:3. 10.3389/fninf.2020.00003. 32116627 10.3389/fninf.2020.00003 PMC7012016

[27] Kivipelto M, Solomon A, Ahtiluoto S, Ngandu T, Lehtisalo J, Antikainen R, et al. The Finnish Geriatric intervention study to prevent cognitive impairment and disability (FINGER): study design and progress. Alzheimers Dement. 2013;9(6):657–65. 10.1016/j.jalz.2012.09.012. 23332672 10.1016/j.jalz.2012.09.012

[28] Krasemann S, Madore C, Cialic R, Baufeld C, Calcagno N, El Fatimy R, et al. The TREM2-APOE pathway drives the transcriptional phenotype of dysfunctional microglia in neurodegenerative diseases. Immunity. 2017;47(3):566-581.e569. 10.1016/j.immuni.2017.08.008. 28930663 10.1016/j.immuni.2017.08.008 PMC5719893

[29] Kurki MI, Karjalainen J, Palta P, Sipilä TP, Kristiansson K, Donner KM, et al. FinnGen provides genetic insights from a well-phenotyped isolated population. Nature. 2023;613(7944):508–18. 10.1038/s41586-022-05473-8. 36653562 10.1038/s41586-022-05473-8 PMC9849126

[30] Leng F, Edison P. Neuroinflammation and microglial activation in Alzheimer disease: where do we go from here? Nat Rev Neurol. 2021;17(3):157–72. 10.1038/s41582-020-00435-y. 33318676 10.1038/s41582-020-00435-y

[31] Lezak M, Howieson D, Bigler E, Tranel D. Neuropsychological assessment. New York: Oxford Univer sity Press; 2012.

[32] Liu CC, Wang N, Chen Y, Inoue Y, Shue F, Ren Y, et al. Cell-autonomous effects of APOE4 in restricting microglial response in brain homeostasis and Alzheimer’s disease. Nat Immunol. 2023;24(11):1854–66. 10.1038/s41590-023-01640-9. 37857825 10.1038/s41590-023-01640-9 PMC11980647

[33] Nguyen AT, Wang K, Hu G, Wang X, Miao Z, Azevedo JA, et al. APOE and TREM2 regulate amyloid-responsive microglia in Alzheimer’s disease. Acta Neuropathol. 2020;140(4):477–93. 10.1007/s00401-020-02200-3. 32840654 10.1007/s00401-020-02200-3 PMC7520051

[34] Okello A, Edison P, Archer HA, Turkheimer FE, Kennedy JC, Bullock R, et al. Microglial activation and amyloid deposition in mild cognitive impairment a PET study. Neurology. 2009. 10.1212/01.wnl.0000338622.27876.0d. 19122031 10.1212/01.wnl.0000338622.27876.0d PMC2817573

[35] Olive C, Ibanez L, Farias FHG, Wang F, Budde JP, Norton JB, et al. Examination of the effect of rare variants in TREM2, ABI3, and PLCG2 in LOAD through multiple phenotypes. J Alzheimers Dis. 2020;77(4):1469–82. 10.3233/jad-200019. 32894242 10.3233/JAD-200019 PMC7927150

[36] Parbo P, Ismail R, Hansen KV, Amidi A, Mårup FH, Gottrup H, et al. Brain inflammation accompanies amyloid in the majority of mild cognitive impairment cases due to Alzheimer’s disease. Brain. 2017;140(7):2002–11. 10.1093/brain/awx120. 28575151 10.1093/brain/awx120

[37] Reiman EM, Chen K, Liu X, Bandy D, Yu M, Lee W, et al. Fibrillar amyloid-β burden in cognitively normal people at 3 levels of genetic risk for Alzheimer’s disease. Proc Natl Acad Sci U S A. 2009;106(16):6820–5. 10.1073/pnas.0900345106. 19346482 10.1073/pnas.0900345106 PMC2665196

[38] Rodriguez GA, Tai LM, Ladu MJ, Rebeck GW. Human APOE4 increases microglia reactivity at Aβ plaques in a mouse model of Aβ deposition. J Neuroinflammation. 2014;11(1):111. 10.1186/1742-2094-11-111. 24948358 10.1186/1742-2094-11-111 PMC4077554

[39] Satoh J-I, Kino Y, Yanaizu M, Tosaki Y, Sakai K, Ishida T, et al. Microglia express ABI3 in the brains of Alzheimer’s disease and Nasu-Hakola disease. Intract Rare Dis Res. 2017;6(4):262–8. 10.5582/irdr.2017.01073. 10.5582/irdr.2017.01073 PMC5735279 29259854

[40] Sekino S, Kashiwagi Y, Kanazawa H, Takada K, Baba T, Sato S, et al. The NESH/Abi-3-based WAVE2 complex is functionally distinct from the Abi-1-based WAVE2 complex. Cell Commun Signal. 2015;13(1). 10.1186/s12964-015-0119-5. 10.1186/s12964-015-0119-5 PMC4589964 26428302

[41] Sims R, Van Der Lee SJ, Naj AC, Bellenguez C, Badarinarayan N, Jakobsdottir J, et al. Rare coding variants in PLCG2, ABI3, and TREM2 implicate microglial-mediated innate immunity in Alzheimer’s disease. Nat Genet. 2017;49(9):1373–84. 10.1038/ng.3916. 28714976 10.1038/ng.3916 PMC5669039

[42] Snellman A, Ekblad LL, Tuisku J, Koivumäki M, Ashton NJ, Lantero-Rodriguez J, et al. APOE ε4 gene dose effect on imaging and blood biomarkers of neuroinflammation and beta-amyloid in cognitively unimpaired elderly. Alzheimers Res Ther. 2023;15(1). 10.1186/s13195-023-01209-6. 10.1186/s13195-023-01209-6 PMC10071691 37016464

[43] Stroop J, Ridley. Studies of interference in serial verbal reactions. J Exp Psychol. 1935;18:643–62.

[44] Therneau T. A package for survival analysis in R. 2024. Retrieved from https://cran.r-project.org/web/packages/survival/vignettes/survival.pdf.

[45] Therneau T, Grambsch P. Modeling survival data: extending the Cox model. New York: Springer; 2000.

[46] Turkheimer FE, Edison P, Pavese N, Roncaroli F, Anderson AN, Hammers A, et al. Reference and target region modeling of [11C]-(R)-PK11195 brain studies. J Nucl Med. 2007;48(1):158–67. 17204713

[47] Ulrich JD, Ulland TK, Mahan TE, Nyström S, Nilsson KP, Song WM, et al. ApoE facilitates the microglial response to amyloid plaque pathology. J Exp Med. 2018;215(4):1047–58. 10.1084/jem.20171265. 29483128 10.1084/jem.20171265 PMC5881464

[48] Verghese PB, Castellano JM, Garai K, Wang Y, Jiang H, Shah A, et al. ApoE influences amyloid-β (Aβ) clearance despite minimal apoE/Aβ association in physiological conditions. Proc Natl Acad Sci U S A. 2013;110(19):E1807-1816. 10.1073/pnas.1220484110. 23620513 10.1073/pnas.1220484110 PMC3651443

[49] Vivash L, O’Brien TJ. Imaging microglial activation with TSPO PET: lighting up neurologic diseases? J Nucl Med. 2016;57(2):165–8. 10.2967/jnumed.114.141713. 26697963 10.2967/jnumed.114.141713

[50] Wechsler D. Wechsler adult intelligence scale - revised, manual. New York: The Psychological Corporation; 1981.

[51] Wechsler D. Wechsler memory scale - revised: manual. San Antonio: The Psychological Corporation; 1987.

[52] Wightman DP, Jansen IE, Savage JE, Shadrin AA, Bahrami S, Holland D, et al. A genome-wide association study with 1,126,563 individuals identifies new risk loci for Alzheimer’s disease. Nat Genet. 2021;53(9):1276–82. 10.1038/s41588-021-00921-z. 34493870 10.1038/s41588-021-00921-z PMC10243600

[53] Yaqub M, Van Berckel BN, Schuitemaker A, Hinz R, Turkheimer FE, Tomasi G, et al. Optimization of Supervised cluster analysis for extracting reference tissue input curves in (R)-[11C]PK11195 brain PET studies. J Cereb Blood Flow Metab. 2012;32(8):1600–8. 10.1038/jcbfm.2012.59. 22588187 10.1038/jcbfm.2012.59 PMC3421099

[54] Yin Z, Rosenzweig N, Kleemann KL, Zhang X, Brandão W, Margeta MA, et al. APOE4 impairs the microglial response in Alzheimer’s disease by inducing TGFβ-mediated checkpoints. Nat Immunol. 2023;24(11):1839–53. 10.1038/s41590-023-01627-6. 37749326 10.1038/s41590-023-01627-6 PMC10863749

[55] Yokokura M, Terada T, Bunai T, Nakaizumi K, Takebayashi K, Iwata Y, et al. Depiction of microglial activation in aging and dementia: positron emission tomography with [11C]DPA713 versus [11C](R)PK11195. J Cereb Blood Flow Metab. 2017;37(3):877–89. 10.1177/0271678X16646788. 27117856 10.1177/0271678X16646788 PMC5363467

[56] Zou J, Tao S, Johnson A, Tomljanovic Z, Polly K, Klein J, et al. Microglial activation, but not tau pathology, is independently associated with amyloid positivity and memory impairment. Neurobiol Aging. 2020;85:11–21. 10.1016/j.neurobiolaging.2019.09.019. 10.1016/j.neurobiolaging.2019.09.019 PMC6919274 31698286

