# Supplementary material for: Genetic validation of ABI3 p.Ser209Phe variant and its effects on early brain pathology in asymptomatic elderly individuals
Source: Alzheimers Res Ther. 2026 Feb 19;18:67. doi: 10.1186/s13195-026-01984-y (PMC13023201; doi:10.1186/s13195-026-01984-y)
Supplement: Supplementary file 1 — Supplementary Material 1. [file 13195_2026_1984_MOESM1_ESM.docx]

## Correlations of [^11^C]PiB, [^11^C]PK11195 and structural imaging variables

| **Table S1. Correlations between imaging variables in the whole sample** | | | | | | | |
| --- | --- | --- | --- | --- | --- | --- | --- |
|  | **Cortex** | **HC** | **PHC** | **ER** | **AMY** | | **PIB** |
| **PIB** | 0.061 | -0.22 | -0.21 | -0.11 | | 0.017 |  |
| **PK** | **0.27*** | 0.061 | 0.029 | -0.059 | | 0.26 | -0,02 |
| Cortex=Cerebral cortex volume, HC=Hippocampus volume, PHC=Parahippocampus volume, ER=Entorhinal volume, Amy=Amygdala volume, PIB=[^11^C]PiB score, PK=[^11^C]PK11195 DVR,* = p < 0.05 | | | | | | | |


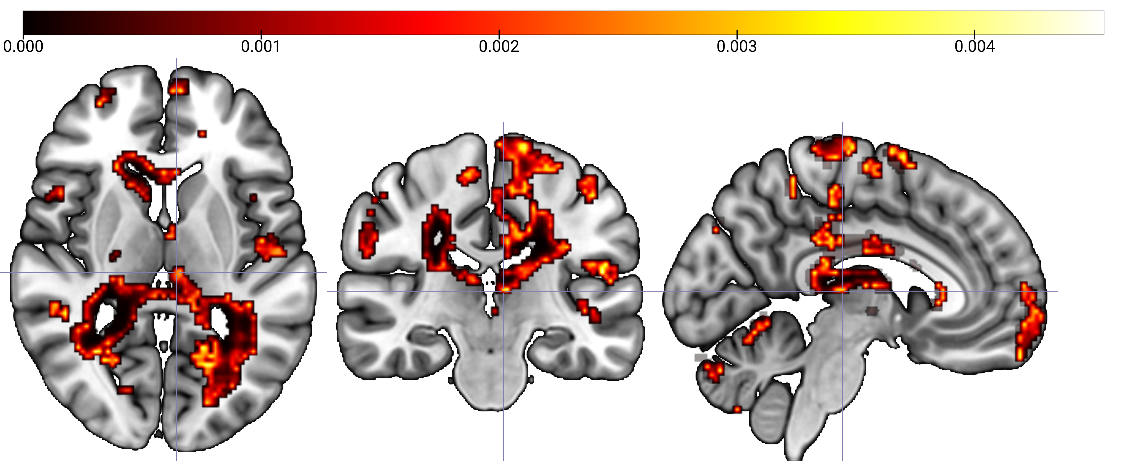


Figure S1 Voxel-level correlations between [^11^C]PiB and [^11^C]PK11195 in the whole sample. The colour scale shows the p-value of the cluster.

## Structural MRI

| Table S2. Volumetric MRI results | | | | | |
| --- | --- | --- | --- | --- | --- |
|  |  | *ABI3^S209F^/ε4* | *ABI3^S209F^/ε3* | *NC* | Group difference |
| Cerebral Cortex Volume | Mean (sd) | 436.6 (34.5) | 425.6 (39.6) | 423.3 (35.7) | 0.30 |
| Entorhinal volume | Mean (sd) | 3.9 (0.6) | 4.0 (0.7) | 3.8 (0.6) | 0.47 |
| Parahippocampal volume | Mean (sd) | 3.9 (0.4) | 3.9 (0.3) | 4.0 (0.6) | 0.50 |
| Amygdala volume | Mean (sd) | 3.1 (0.4) | 3.2 (0.5) | 3.0 (0.5) | 0.57 |


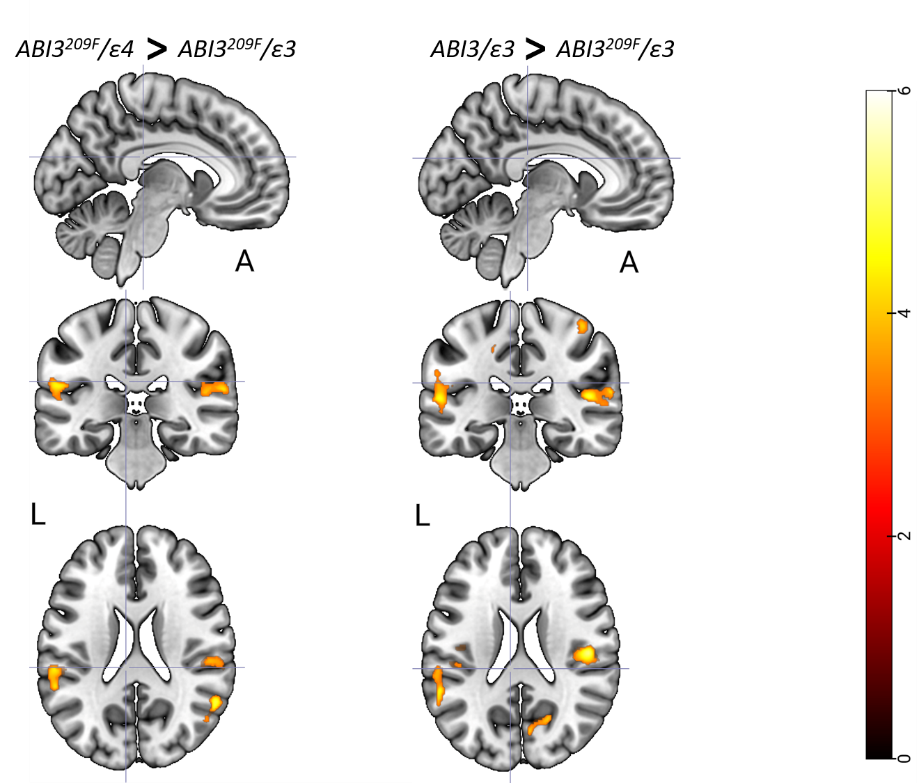


Figure S2 ABI3^S209F^/ε3 group had lower grey matter volume than the ABI3^S209F^/ε4 and NC. FDR corrected p<0.001 in red-yellow.
